# α-spinasterol isolated from *Achyranthes aspera* L. ameliorates inflammation via NF-κB and Nrf2/HO-1 pathways

**DOI:** 10.1038/s41598-025-90022-2

**Published:** 2025-02-17

**Authors:** Qiongli Zeng, Weiting Xiao, Heng Zhang, Wei Liu, Xionglong Wang, Zhen Li, Yue Han, Zhi Wang, Shunxiang Li, Jinwei Yang, Wen Ouyang

**Affiliations:** 1 School of Pharmacy, Hunan University of Chinese Medicine, Changsha, 410208 China; 2 Key Laboratory of Modern Research of TCM, Education Department of Hunan Province, Changsha, 410208 China; 3 School of Integrated Chinese and Western Medicine, Hunan University of Chinese Medicine, Changsha, 410208 China; 4 Analysis of Complex Effects of Proprietary Chinese Medicine, Hunan Provincial Key Laboratory, Yongzhou, 410116 China; 5 The Second Hospital of Integrated Chinese and Western Medicine Affiliated to Hunan University of Chinese Medicine, Liuyang, 410300 China

**Keywords:** α-Spinasterol, Content determination, NF-κB, Anti-inflammatory, Nrf2, Biochemistry, Plant sciences, Medical research

## Abstract

On the basis of previous studies, the low-polar part of *Achyranthes aspera* L. (*A. aspera*) had strong anti-inflammatory activity. Three compounds were isolated from the low polarity fraction of *A. aspera*, and their structures were identified as α-spinasterol (**1**), 7,8-dihydrospinasterol (**2**), 22,23-dihydrospinasterol (**3**). Among them, the content of α-spinasterol (**1**) in *A. aspera* was higher in the spring and winter seasons through HPLC methods, ranging from 0.0085 to 0.0157%. Futhermore, in the LPS-induced RAW264.7 cells inflammation model, α-spinasterol significantly reduced the levels of cytokines such as IL-6, PGE2 and TNF-α, inhibited the expression of COX-2, 5-LOX, p-IKKβ, p-NFκB and p-IkBα proteins, and promoted the expression of Nrf2, HO-1 and NQO1 proteins. Therefore, this study showed that α-spinasterol can inhibit LPS-induced RAW264.7 cells inflammation, and its mechanism may be related to the inhibition of NF-κB pathway, activation of Nrf2 pathway, and reduction of excessive release of inflammatory factors.

## Introduction

Inflammation is a defense response of living tissues with vascular system to various injury factors^1^. The occurrence and development of most diseases are accompanied by inflammatory processes, such as cancer, diabetes, arthritis, cardiovascular diseases, Alzheimer’s disease, and neurological diseases^2–4^. As a type of immune cell, macrophages play a critical role in inflammation, safeguarding the body against external invasion by producing inflammatory cytokines, including TNF-α, IL-6, and IL-1β^5–7^. Lipopolysaccharides (LPS) are an inflammatory inducer that can activate macrophages. Therefore, the LPS induced RAW264.7 cells inflammation model is considered a classic anti-inflammatory drug screening model and widely used in vitro anti-inflammatory research^8^.

The NF-κB signaling pathway is typically activated by LPS, TNF-α, IL-1β, among other factors. This activation results in the degradation of IκB, facilitating the release and subsequent nuclear translocation of the NF-κB p65/p50 dimer. This translocation triggers the transcriptional activation of target genes, such as COX-2, thereby initiating a cascade of inflammatory stress events^9,10^. Due to this, inhibiting the NF-κB signaling pathway holds significant therapeutic potential for the treatment of inflammatory diseases. Recent studies have demonstrated that Nrf2 has a cross effect with the NF-κB pathway. Activating the Nrf2 pathway not only has an antioxidant stress effect, but also weakens the stimulation of pro-inflammatory cytokines on cells and tissues, prevents the generation of a large amount of reactive oxygen species (ROS) in cells, and avoids the vicious cycle of further activating NF-κB to produce a large amount of inflammatory factors^11,12^.

*Achyranthes aspera* L. (*A. aspera*) is a herbaceous plant and belong to the family Amaranthaceae, also called Tuniuxi in China. In traditional Chinese medicine, Tuniuxi is extensively used for treatment of traumatic injury, rheumatic joint pain, dysentery, diphtheria, sore throat, and diabetes mellitus^13–15^. Studies have demonstrated that various polar fractions of *A. aspera* have NO inhibitory effect on LPS-induced RAW264.7 cells, whereas the low polarity fraction displays the most pronounced inhibitory activity^16^. Thus, we focused on isolating the low-polarity components of *A. aspera*, resulting in the identification of three compounds: α-spinasterol (**1**), 7,8-dihydrospinasterol (**2**), and 22,23-dihydrospinasterol (**3**). It should be noted that α-spinasterol was found to be the highest content among three compounds.

α-spinasterol, a naturally-occurring steroid, has a cyclopentane-fused polyhydrophenanthrene structure as its fundamental framework, with a hydroxyl group located at the C_3_ position, resembling cholesterol^17^. Modern pharmacological studies have confirmed that α-spinasterol has rich pharmacological effects such as anti-inflammatory, anti-tumor, anti-convulsion, anti-anxiety and anti-depression^18–21^. It is safe and non-toxic, and is a phytosterol with significant nutritional value and promising application.

In order to understand the physiological role of α-spinasterol in more detail, we are particularly interested in its anti-inflammatory properties. The present study focuses on α-spinasterol isolated from *A. aspera* as the research subject, aiming to investigate its potential anti-inflammatory effects by inhibiting oxidative stress and regulating immunity through the modulation of target proteins involved in the interaction between Nrf2 and NF-κB signaling pathways. Addressing this issue may offer novel insights for the advancement and utilization of α-spinasterol and *A. aspera*.

## Results

### Compounds identification

**Fig. 1:**
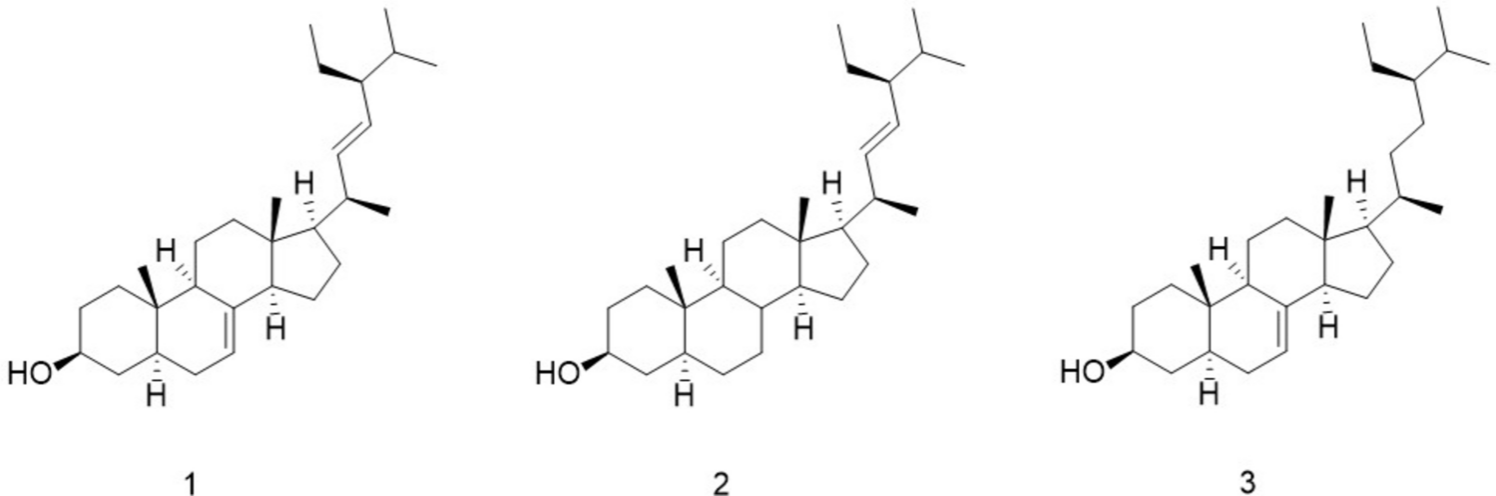
The chemical structures of three compounds (**1**: α-spinasterol, **2**: 7,8-dihydrospinasterol, **3**: 22,23-dihydrospinasterol).

Three compounds (Fig. 1) were isolated from the low-polar fractions of *A. aspera* for the first time, and identified as α-spinasterol (**1**), 7,8-dihydrospinasterol (**2**), 22,23-dihydrospinasterol (**3**) through data comparison with literatures^22–24^. In the process of using semi-preparative high performance liquid chromatography separation (Semi-Prep HPLC), we utilized an ODS column characterized by a high affinity for low polar compounds. Additionally, we incorporated a small volume of isopropanol into acetonitrile as the mobile phase to optimize the separation of the three target compounds.

Compound **1** was obtained as white needle like crystals. Analysis of its ^1^H-NMR data revealed one oxymethine proton *δ*_H_ 3.62 (1H, m), and two olefinic protons [*δ*_H_ 5.05 (1H, dd, *J* = 8.8, 15.1 Hz), 5.18 (2H, overlapped)]. Combined with ^13^C-NMR data showed 29 carbon signals characteristic for cholesterol-type steroids skeleton, including six methyl groups (*δ*_C_ 12.1, 13.1, 19.0, 12.3, 21.1, 21.4), four olefinic carbons (*δ*_C_ 117.5, 139.6, 138.2, 129.4) form two double bonds, one highly deshielded oxygenated methine (*δ*_C_ 71.1 and *δ*_H_ 3.62) repectively. Comparing the ^1^H and ^13^C NMR results with the literature, the compound was identified as α-spinasterol, and further confirmed by high performance liquid chromatography (HPLC) comparison with α-spinasterol standard. The ^1^H and ^13^C NMR data are assigned as follows. ^1^H-NMR (CDCl_3_, 600 MHz) *δ* (ppm): 0.57 (3H, s, H-18), 0.83 (9H, overlapped, H-19, 26, 29), 0.87 (3H, d, *J* = 6.2 Hz, H-27), 1.05 (3H, d, *J* = 6.6 Hz, H-21), 3.62 (1H, m, H-3), 5.05 (1H, dd, *J* = 8.8, 15.1 Hz, H-23), 5.18 (2H, m, H-7, 22); ^13^C-NMR (CDCl_3_, 150 MHz) *δ* (ppm): 37.2 (C-1), 31.5 (C-2), 71.1 (C-3), 38.0 (C-4), 40.3 (C-5), 29.7 (C-6), 117.5 (C-7), 139.6 (C-8), 49.5 (C-9), 34.2 (C-10), 21.6 (C-11), 39.5 (C-12), 43.4 (C-13), 55.1 (C-14), 23.0 (C-15), 28.5 (C-16), 55.9 (C-17), 12.1 (C-18), 13.1 (C-19), 40.8 (C-20), 21.4 (C-21), 138.2 (C-22), 129.4 (C-23), 51.3 (C-24), 31.9 (C-25), 19.0 (C-26), 21.1 (C-27), 25.4 (C-28), 12.3 (C-29).

Similar to compound **1**, compound **2** also had six methyl groups [*δ*_H_ 0.67 (3H, s) 0.83 (9H, overlapped), 0.88 (3H, d), 1.03 (3H, d)], one oxymethine proton *δ*_H_ 3.61 (1H, m). The difference was that two olefinic protons [*δ*_H_ 5.04 (1H, dd, *J* = 15.2 Hz, *J* = 8.8 Hz), 5.15 (1H, dd, *J* = 15.2 Hz, *J* = 8.7 Hz) undergo a coupling reaction to form a trans-double bond, and according to ^13^C data (*δ*_C_ 138.4, 129.2), it is inferred that there is only one double bond of compound **2**, which is distributed in C-22 and C-23 like α-spinasterol. Compared with the literature data, compound **2** was identified as 7,8-dihydrospinasterol, and its detailed data distribution is as follows. ^1^H-NMR (CDCl_3_, 600 MHz) *δ* (ppm): 0.67 (3H, s, H-18), 0.83 (9H, overlapped, H-19, 26, 29), 0.88 (3H, d, *J* = 6.4 Hz, H-27), 1.03 (3H, d, *J* = 6.6 Hz, H-21), 3.61 (1H, m, H-3), 5.04 (1H, dd, *J* = 15.2 Hz, *J* = 8.8 Hz, H-23), 5.15 (1H, dd, *J* = 15.2 Hz, *J* = 8.7 Hz, H-22); ^13^C-NMR (CDCl_3_, 150 MHz) *δ* (ppm): 37.0 (C-1), 31.6 (C-2), 71.4 (C-3), 38.2 (C-4), 44.9 (C-5), 28.9 (C-6), 32.1 (C-7), 35.5 (C-8), 54.4 (C-9), 35.4 (C-10), 21.2 (C-11), 39.9 (C-12), 42.5 (C-13), 56.6 (C-14), 24.3 (C-15), 28.7 (C-16), 56.1 (C-17), 12.2 (C-18), 12.2 (C-19), 40.5 (C-20), 21.3 (C-21), 138.4 (C-22), 129.2 (C-23), 51.3 (C-24), 31.9 (C-25), 18.9 (C-26), 21.1 (C-27), 25.4 (C-28), 12.3 (C-29).

Similarly, the ^1^H and ^13^C NMR data for compound **3** closely resembled those of compound **1** and **2**. The signals at *δ*_H_ 5.18 (1H, m) and *δ*_C_ 117.4, 139.6 indicated that the sole double bond of compound **3** was located at C-7 and C-8. Upon comparison with existing literature, the compound was identified as 22,23-dihydrospinasterol. The detailed distribution of the data is presented below. ^1^H-NMR (CDCl_3_, 600 MHz) *δ* (ppm): 0.56 (3H, s, H-18), 0.70 (3H, s, H-19), 0.85 (6H, overlapped, H-26, 29), 1.03 (3H, d, *J* = 6.6 Hz, H-21), 3.58 (1H, m, H-3), 5.18 (1H, m, H-7); ^13^C-NMR (CDCl_3_, 150 MHz) *δ* (ppm): 37.2 (C-1), 31.5 (C-2), 71.1 (C-3), 38.0 (C-4), 40.3 (C-5), 29.2 (C-6), 117.4 (C-7), 139.6 (C-8), 49.5 (C-9), 34.2 (C-10), 21.6 (C-11), 39.6 (C-12), 43.4 (C-13), 55.1 (C-14), 23.0 (C-15), 28.0 (C-16), 56.1 (C-17), 11.9 (C-18), 13.1 (C-19), 36.6 (C-20), 18.9 (C-21), 33.9 (C-22), 26.2 (C-23), 45.8 (C-24), 29.7 (C-25), 19.8 (C-26), 19.4 (C-27), 23.1 (C-28), 11.9 (C-29) (Supplementary file 1).

### Content determination of α-spinasterol

**Fig. 2:**
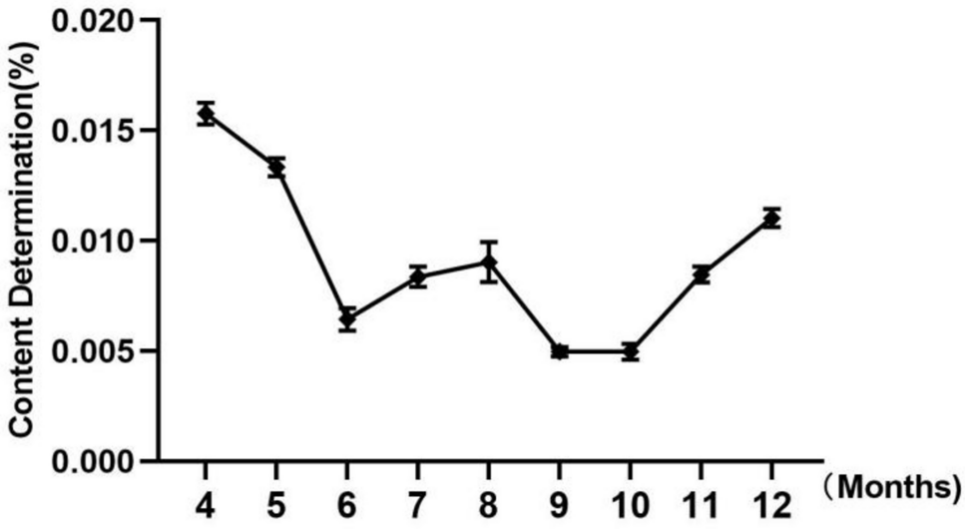
The content change of α-spinasterol in *A. aspera* with seasonal harvest.

In this experiment, α-spinasterol was used as an index, and its content in *A. aspera* harvested in different months was determined by HPLC methods, providing a reference for the planting and harvesting of *A. aspera*. Fig. 2 illustrated that the α-spinasterol content in *A. aspera* varies significantly across different months. Specifically, in April, when the aerial parts of *A. aspera* not emerged, the α-spinasterol content reached its peak at 0.0157%. Conversely, during the fruiting period in September and October, the content was at its lowest, measuring 0.005%. Furthermore, the mean α-spinasterol content of *A. aspera* from April to December was at 0.0091%. Based on these results, we found that the content of α-spinasterol in *A. aspera* was higher in spring and winter.

### Effect of α-spinasterol on cell viability in RAW264.7 cells

**Fig. 3:**
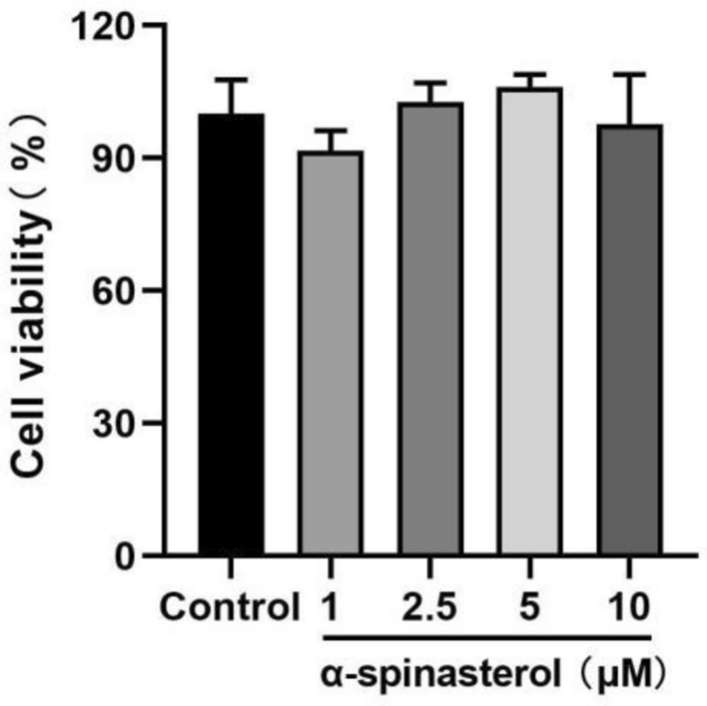
The effects of α-spinasterol on cell viability in RAW264.7 cells.

Initially, we assessed the cytotoxicity of α-spinasterol in RAW264.7 cells to determine the concentration of α-spinasterol for anti-inflammatory analysis. As shown in Fig. 3, CCK8 assay was used to evaluate the effect of α-spinasterol on cell viability in RAW264.7 cells. The results showed that it was non-toxic to RAW264.7 cells in the concentration range of 1–10 μM. However, concentrations exceeding 10 μM resulted in diminished cell adhesion and increased cell detachment.

### Effect of α-spinasterol on NO production in RAW264.7 cells

**Fig. 4:**
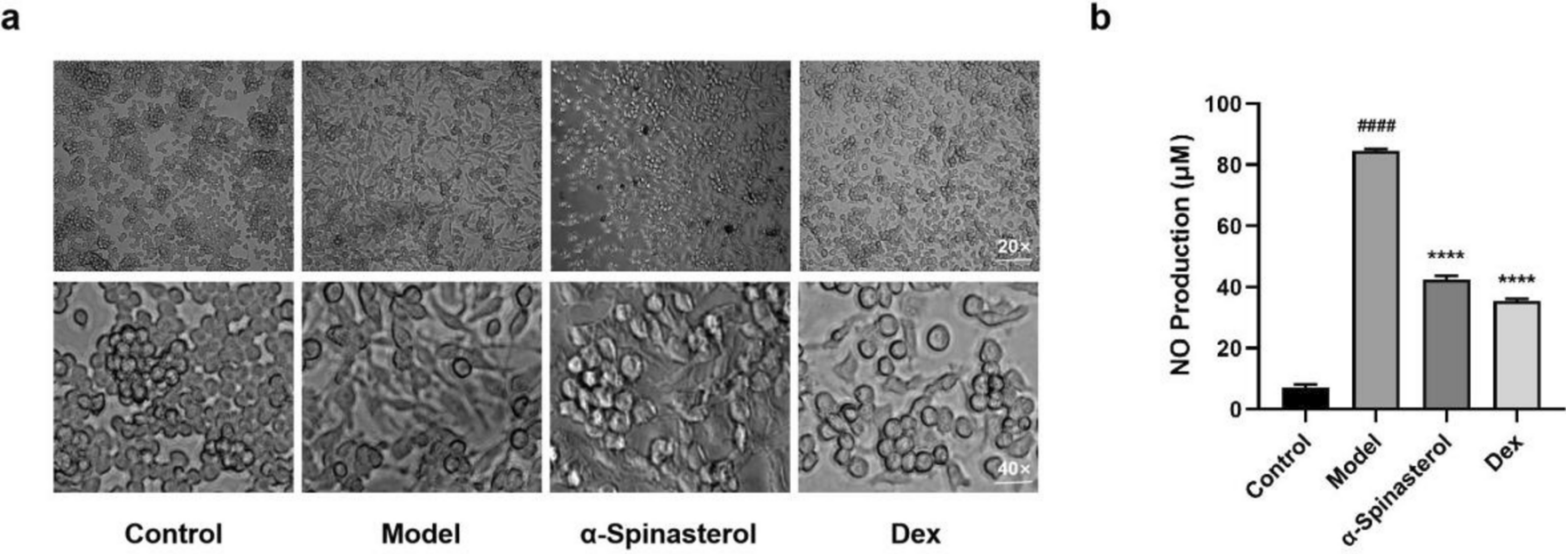
(**a**) Morphological observation of RAW264.7 cells treated with α-spinasterol; (**b**) The effects of α-spinasterol on NO production in LPS-Induced RAW264.7 cells (^####^*p* < 0.0001 vs. Control group; ****p < 0.0001 vs. Model group).

Induced by LPS, RAW264.7 cells differentiate into M1 type cells and produce pro-inflammatory cytokines^25^. To preliminarily assess the anti-inflammatory effects of α-spinasterol, cells were induced with 1 μg/mL LPS to create a model^26^, as well as intervention with α-spinasterol or positive drug dexamethasone (Dex) respectively. Research indicated that α-spinasterol exhibited a dose-dependent inhibitory effect on NO release ranging from 1 to 5 μM, with optimal inhibition concentration at 5 μM. As shown in Fig. 4, compared with the control group, the NO content in the cell supernatant of the model group was significantly increased (*p* < 0.0001). Compared with the model group (1 μg/mL LPS), the NO content in both Dex group and α-spinasterol group was markedly reduced (*p* < 0.0001). Additionally, under the microscope, RAW264.7 cells appear to be long spindle, diamond or irregular after LPS stimulation, as well as having increased volume and pseudopods. Compared with the model group, the cells treated with α-spinasterol (5 μM) and Dex (10 μg/mL) showed a more normal volume and a reduction in pseudopods.

### Effect of α-spinasterol on pro-inflammatory cytokines production in LPS-induced RAW264.7 cells

**Fig. 5:**
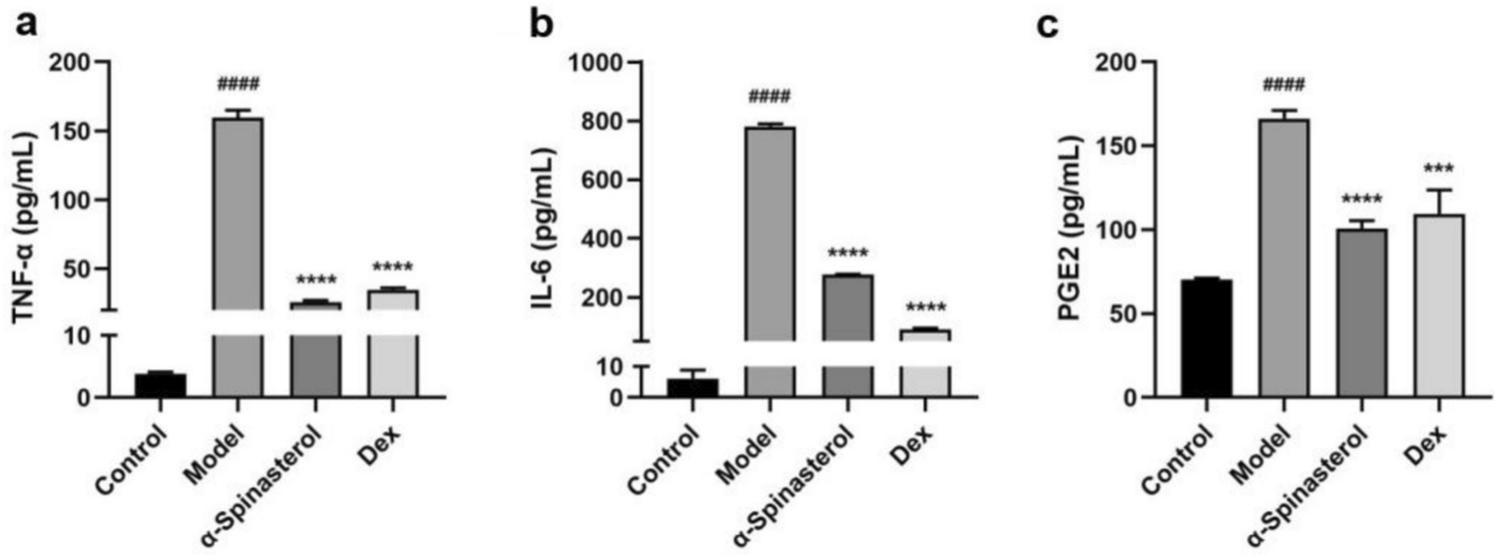
The effects of α-spinasterol on TNF-α, IL-6 and PGE2 levels in LPS-Induced RAW264.7 cells ((**a**) TNF-α, (**b**) IL-6, (**c**) PGE2). Results are shown as the mean ± SD (*n* = 3). ^####^*p* < 0.0001 vs. Control group; ***p < 0.001, ****p < 0.0001 versus Model group.

To examine α-spinasterol’s anti-inflammatory activity further, we examined its effects on RAW264.7 cells induced with LPS to produce TNF-α, IL-6, and PGE2. The results of the experiment are presented in Fig. 5, which depicts the concentration of inflammatory factors within the cell supernatant (pg/mL). The levels of TNF-α, IL-6, and PGE2 in the cell supernatant of the model group were significantly elevated compared to the control group (*p* < 0.0001), thereby confirming the successful establishment of an LPS-induced inflammatory model in RAW264.7 cells. Treatment with α-spinasterol and Dex resulted in a significant reduction in the concentrations of TNF-α, IL-6, and PGE2 relative to the model group (*p* < 0.001).

### α-Spinasterol’s effects on proteins expression of COX-2 and 5-LOX

**Fig. 6:**
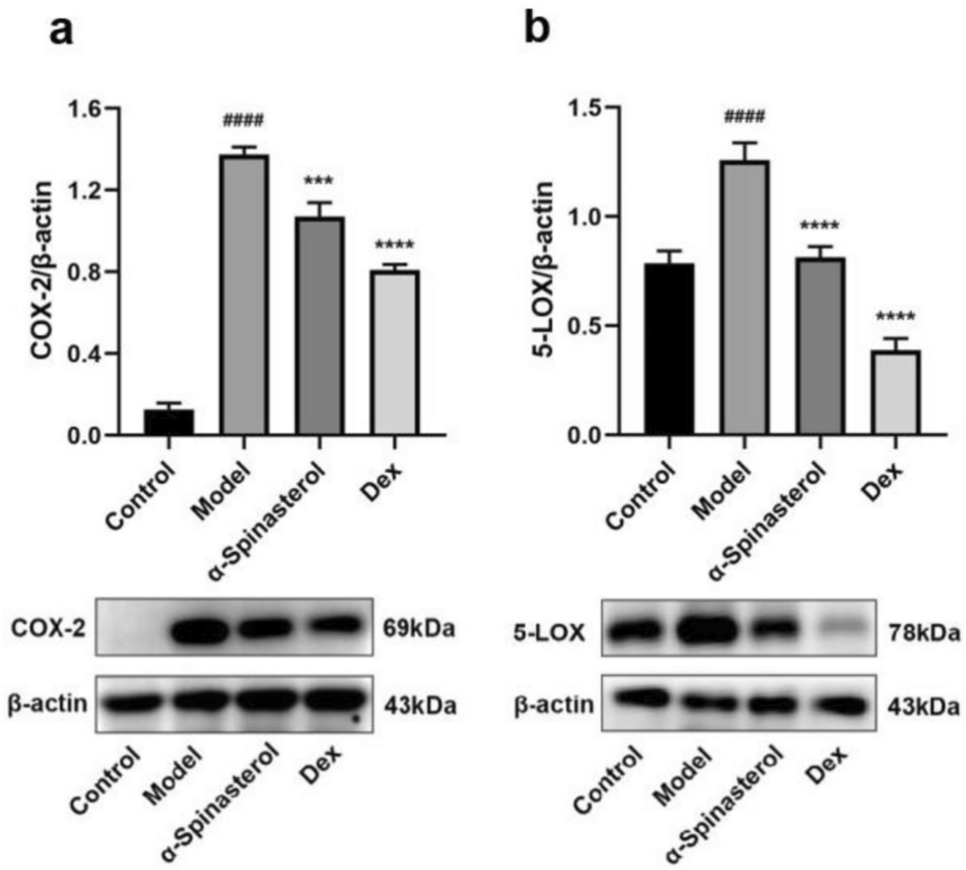
(**a**) Quantification and expression of COX-2. (**b**) Quantification and expression of 5-LOX. Results are shown as the mean ± SD (*n* = 3). ^####^*p* < 0.0001 versus Control group; ***p < 0.001, ****p < 0.0001 versus Model group.

COX-2 and 5-LOX are the fundamental regulators of the inflammatory process, and their inhibition is considered an effective strategy for preventing inflammatory diseases^27^. The effects of α-spinasterol on COX-2 and 5-LOX proteins expression were studied by Western Blot. As illustrated in Fig. 6, the expression levels of COX-2 and 5-LOX proteins in the model group (1 μg/mL LPS) were significantly elevated (*p* < 0.0001). Conversely, treatment with α-spinasterol and Dex resulted in a significant reduction in the expression of COX-2 and 5-LOX proteins compared to the model group (*p* < 0.001). The results suggested that α-spinasterol may inhibit inflammation through concurrent suppression of COX-2 and 5-LOX expression.

### α-Spinasterol’s effects on proteins expression of p-IKKβ, p-NF-κB and p-IkBα

**Fig. 7:**
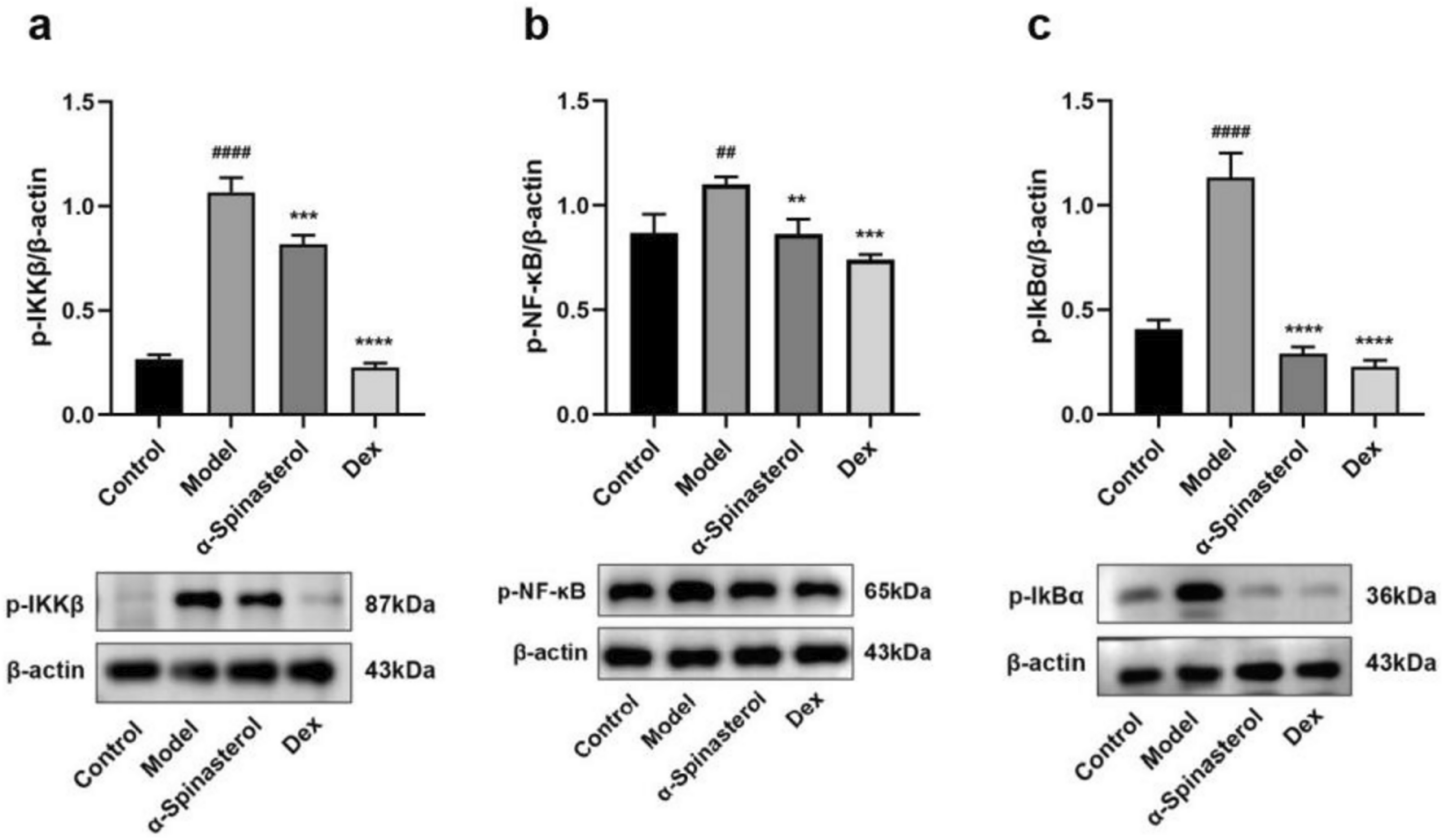
(**a**) Quantification and expression of p-IKKβ. (**b**) Quantification and expression of p-NF-κB. (**c**) Quantification and expression of p-IkBα. Results are shown as the mean ± SD (*n* = 3). ^##^*p* < 0.01, ^####^*p* < 0.0001 versus Control group; **p < 0.01, ***p < 0.001, ****p < 0.0001 versus Model group.

As an important transcription factor, NF-κB regulates the production of pro-inflammatory mediators in activated macrophages^28^. Consequently, the potential of α-spinasterol to inhibit NF-κB signaling pathway was evaluated. Compared with the control group, the expression levels of p-IKKβ, p-NF-κB and p-IkBα proteins in the model group (1 μg/mL LPS) were significantly increased (*p* < 0.01). Compared with the model group, the expressions levels of p-IKKβ, p-IkBα and p-NF-κB proteins in the cells treated with α-spinasterol were significantly reduced (*p* < 0.01). This suggested that α-spinasterol may exert an anti-inflammatory effect by inhibiting the activation of proteins associated with the NF-κB signaling pathway. Similarly, the positive drug, Dex, effectively inhibited the expression of NF-κB pathway-related proteins (details see Fig. 7).

### α-Spinasterol’s effects on proteins expression of Nrf2, HO-1 and NQO1

**Fig. 8:**
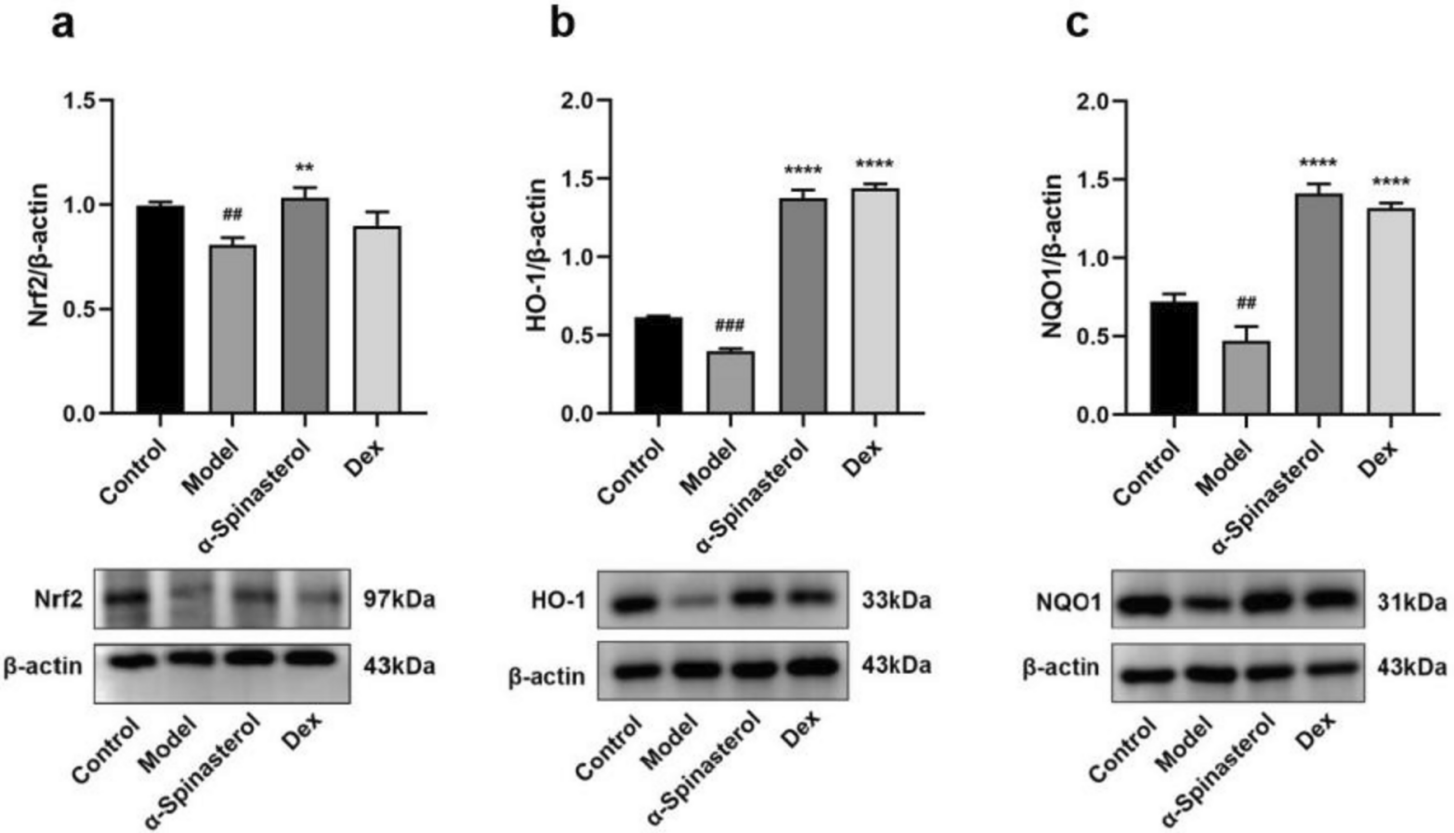
(**a**) Quantification and expression of Nrf2. (**b**) Quantification and expression of HO-1. (**c**) Quantification and expression of NQO1. Results are shown as the mean ± SD (*n* = 3). ^##^*p* < 0.01, ^###^*p* < 0.001 versus Control group; **p < 0.01, ****p < 0.0001 versus Model group.

Nrf2 is a pivotal transcription factor involved in the cellular antioxidant stress response, with downstream targets including antioxidant factors such as HO-1 and NQO1^29^. Activation of the Nrf2 signaling pathway can exert antioxidant and anti-inflammatory effects, so the regulation of α-spinasterol on Nrf2, HO-1 and NQO1 proteins was evaluated by Western Blot analysis. According to Fig. 8, the expression levels of Nrf2, NQO1 and HO-1 proteins significantly diminished in the model group (1 μg/mL LPS) compared to the control group (*p* < 0.01). Compared with the model group, the α-spinasterol group exhibited a significant upregulation in the expression of NQO1, HO-1 and Nrf2 proteins (*p* < 0.01). This indicated that α-spinasterol may exert anti-inflammatory activity by activating the expression of Nrf2, HO-1 and NQO1 proteins to regulate cell redox. At the same time, the Dex group demonstrated a significant increase in the levels of HO-1 and NQO1 proteins (*p* < 0.0001).

## Discussion

For many years, *A. aspera* has been widely used around the world as a herbal medicine due to its rich use history and extensive efficacy. However, prior research on the chemical constituents of *A. aspera* has predominantly concentrated on saponins and steroidal ketones. Considering the traditional application of *A. aspera* in China, where it is finely powdered and administered to the throat for the treatment of tonsillitis and pharyngolaryngitis, we hypothesize that the low-polarity components of *A. aspera*, which are not readily extracted by water, may also possess substantial anti-inflammatory potential. Moreover, in previous studies, we assessed the anti-inflammatory properties of various polarity fractions and discovered that the acetone fraction demonstrated the most potent anti-inflammatory activity^16^. Therefore, this study aimed to investigate the low-polarity constituents of *A. aspera* to enhance the understanding of its chemical composition and to provide a scientific foundation for its clinical application.

In this paper, three compounds α-spinasterol (**1**), 7,8-dihydrospinasterol (**2**), 22,23-dihydrospinasterol (**3**) were isolated from the low polar components of *A. aspera* for the first time. Furthermore, we previously isolated and identified over 30 compounds from *A. aspera*, including saponins, steroidal ketones, alkaloids, and isoflavones^16^. Among these, α-spinasterol demonstrated superior anti-inflammatory activity compared to the other compounds, prompting us to undertake a systematic investigation. Initially, we analyzed the content of α-spinasterol in *A. aspera* across different months. The findings indicated that the α-spinasterol concentrations peaked in April when *A. aspera*'s aerial parts not emerged from the ground. During the flowering phase, from June to August, there was an upward trend in α-spinasterol content. Conversely, during the fruiting period, from September to October, α-spinasterol content reached its lowest point, and the content increased significantly from November to December when the aboveground part withered. Overall, the variation in α-spinasterol content aligned into the growth dynamics of *A. aspera*, offering valuable insights for the scientific cultivation and harvest practices of this medicinal plant.

In vitro experiments demonstrated that stimulation of RAW264.7 cells with LPS for 24 h resulted in a significant increase in NO levels in the cell supernatant. Co-treatment with α-spinasterol and LPS for the same duration significantly inhibited NO production in the cell supernatant, suggesting that α-spinasterol possesses anti-inflammatory properties. Furthermore, ELISA results indicated that the levels of TNF-α, IL-6 and PGE2 in the cell supernatant were significantly reduced under α-spinasterol intervention compared to the model group. Western Blot analysis revealed a decrease in COX-2, 5-LOX, p-IKKβ, p-NF-κB and p-IkBα proteins expression and an increase in Nrf2, HO-1 and NQO1 proteins expression relative to the model group.

5-LOX and COX-2, as fundamental regulators of inflammatory processes, are key metabolic enzymes in the arachidonic acid metabolic pathway and have been recognized as effective ways to prevent acute inflammatory diseases^30^. Tissue damage activates the trimer IKK complex via an intracellular signaling cascade, which then phosphorylates I-κBα, leading to phosphorylated NF-κB nuclear translocations that further activate downstream inflammatory and chemokines^31^. Nrf2 can play an antioxidant role by regulating the expression of NQO1 and HO-1^32^. Meanwhile, there is crosstalk among arachidonic acid metabolism, NF-κB entry into the nucleus and Nrf2/HO-1 signaling pathways. Salidroside improved gouty arthritis by reprogramming COX-2 and 5-LOX-mediated arachidonic acid metabolism and by influencing NF-κB signaling to polarize macrophages away from the M1 phenotype^33^. Peptides have been reported to alleviate oxidative stress and inflammation by synergistically regulating Nrf2/HO-1 and NF-κB signaling pathways^34^_._ α-spinasterol was firstly found to play an anti-inflammatory role through regulating the arachidonic acid metabolic pathway, NF-κB nucleation and Nrf2 signaling pathways in the present research. In conclusion, this study offers valuable insights for the research and development of *A. aspera* and its active constituent, α-spinasterol, in the treatment of associated inflammatory diseases.

## Materials and methods

### Plant material and reagents

*Achyranthes aspera* L. (*A. aspera*) was provided by Hunan Times Sunshine Pharmaceutical Co., Ltd. Medicinal Planting Base (Yongzhou, China). It was identified and authenticated by Dr. Zhi Wang, Research Officer, Department of Pharmaceutical Botany, Hunan University of Chinese Medicine (Changsha, China). A voucher specimen (ID: 2018072202) has been preserved in the Engineering Technology Research Center for Screening Active Substances of Traditional Chinese Medicine, Hunan University of Chinese Medicine. This study complies with the experimental research regulations of Hunan University of Chinese Medicine.

α-spinasterol (> 98%) was procured from Chengdu Push Bio-technology Co., Ltd. (Chengdu, China); silica-gel plate GF254, 80–200 mesh column chromatography silica gel was procured from Qingdao Hailang silica gel desiccant Co., Ltd. (Qingdao, China); acetonitrile, methanol, and reverse phase silica gel plate were procured from German Merck KGaA (Darmstadt, Germany); ODS-A spherical filler was procured from YMC Company (Kyoto, Japan). All other chemicals and reagents used for the study were of analytical grade procured from Tianjin zhi yuan Chemical Reagent Co., Ltd. (Tianjin, China).

### Extraction procedure

The shade-dried roots (1.17 kg) of *A. aspera* were powered and extracted three times with 12 L methanol (MeOH) for 2 h at 60 °C. The combined solution evaporated to dryness by rotary evaporator at 60 °C under decreased pressure to obtain 130 g crude extract.

### Isolation and identification

The crude extract (130 g) was fractionated by ODS-AQ reverse-phase (RP) silica gel column (117.5 g, 4 cm × 10 cm) chromatography eluting with MeOH-H_2_O (0: 100, 1: 9, 3: 7, 1: 1, 7: 3, 9: 1, 100: 0, *v/v*) and acetone to obtain eight fractions (Fr. A-H) based on TLC analyses. Fr. H (1.27 g) was separated by silica gel column (100–200 mesh, 10 cm × 60 cm) and eluted with petroleum ether-ethyl acetate (10: 0–0: 10, *v/v*) to give fifteen subfractions (Fr. H1-15). Fr. H2 was concentrated to obtain 150 mg crystals, which were further separated by semi-preparative RP-HPLC with the mobile phase of acetonitrile-isopropyl alcohol (2.1: 0.9, *v/v*, 3 mL/min) to afford compound **1**, compound **2** and compound **3** respectively.

Finally, the structures of isolated compounds were identified by the combination of NMR techniques or compared with the reference substances. NMR: Bruker Avance-600 spectrometer; at 600 (^1^H) and 150 (^13^C) MHz; residual solvent peaks as internal standard; *δ* in ppm and* J* in Hz.

### Content determination of α-spinasterol by HPLC

#### Preparation of standard and sample solutions

Standard solution: accurately weigh 5 mg of α-spinosterol, dissolve it in MeOH, and dilute it to achieve a final concentration of 0.5 mg/mL.

Sample solution: extract 4.0084 g of *A. aspera* root powder twice with 50 mL cyclohexane using heating reflux. dry the extract using a rotary evaporator at 60 °C, and then dilute to 5 mL with MeOH. Then, filter with a 0.45 μm polypropylene filter and transfer to HPLC glass vials.

#### HPLC chromatographic condition

HPLC data were recorded on a Shimadzu LC-20AT HPLC instrument equipped with ODS-C_18_ column (4.5 mm × 250 mm, 5 μm). The HPLC conditions were as follows: mobile phase A acetonitrile and mobile phase B isopropanol (9: 1, *v/v*), the flow rate was 1.0 mL/min; the column temperature was 30 °C; the detection wavelength was at 205 nm; and the injection volume was 20 μL.

### Method validation

The linearity of the calibration curves was determined by plotting the peak area (Y) of the analytes against their concentrations (X). The standard curve equations of the α-spinasterol were within the linear range of 0.025–0.5 mg/mL, with both *R*^2^ greater than 0.999, indicating that the method was good. To assess instrument precision, 20 μL of the reference solution was injected six times under chromatographic conditions, yielding a peak area (Relative standard deviation) RSD of 0.86% for α-spinasterol, indicating high precision. Six parallel sample solutions were then prepared and analyzed similarly, resulting in a content RSD of 1.76% for α-spinasterol, demonstrating good method repeatability. To evaluate analyte stability, a sample solution was stored at 4 °C and tested at intervals of 0, 2, 4, 8, 12, and 24 h using chromatography. The RSD value for α-spinasterol peak area was 1.71%, showing stability over 24 h. Six sample solution with known content were mixed with a reference solution in a specific ratio. After thorough mixing, the samples were analyzed using HPLC. The recovery rate is calculated according to the following formula: recovery rate (%) = (detection amount-original amount)/addition amount × 100%. The average recovery of α-spinasterol was 98.95%, with an RSD of 0.81%, indicating the HPLC method’s reliability.

### Cell culture

RAW264.7 cell, a mouse macrophage cell line, was procured from Procell Life Science & Technology Co., Ltd. (Wuhan, China), and cultured in DMEM solution that contained 10% fetal bovine serum (FBS), 100 U/mL penicillin, and 100 μg/mL streptomycin in a humidified environment at 37 °C with 5% CO_2_.

### Cell viability assay

Cell viabilities were determined using the CCK8 method^35^. In brief, RAW264.7 cells (8 × 10^4^ cells per well) were seeded into 96-well plates and incubated overnight, then various concentrations of the α-spinasterol (1, 2.5, 5, 10 μM) were added to each cell plate. After 24 h incubation, CCK8 (10%) solution was added and further incubated at 37 °C for 1 h, and the absorbance was determined at 450 nm. All assays were performed in triplicate.

### NO release assay

The inhibitory effect of compounds on NO production in RAW264.7 cells was evaluated by Griess method according to previous reports^36^. Briefly, RAW264.7 cells were seeded in 6-well plates and induced with LPS (1 μg/mL) in complete DMEM supplemented with α-spinasterol (1, 2.5, 5 μM) or Dex (10 μg/mL) for 24 h. After 24 h, the 100 μL supernatants and Griess reagent (Shanghai Yuanye Bio-Technology Co., Ltd, Shanghai, China) were mixed at 37 °C, absorbance was determined at 540 nm using a microplate reader. All assays were performed in triplicate.

### ELISA of TNF-α, PGE2 and IL-6

After overnight culture in a 12-well plate (4 × 10^5^ cells/mL), the cells were treated with α-spinasterol (5 μM) and Dex (10 μg/mL) plus LPS (1 μg/mL) for 24 h, the levels of TNF-α, PGE2 and IL-6 in the cell supernatant were measured by ELISA kit (Proteintech, Wuhan, China) according to the manufacturer 's instructions. The OD value was then measured by a microplate reader (Bio-Rad, california, USA) at 450 nm, quantified by a standard curve, and analyzed by Graph Pad prism 8.0 software.

### Western blot analysis

The RAW264.7 cells (8 × 10^5^ cells/well) in a 6-well culture plate were subjected to treatment with 5 μM α-spinasterol and 10 μg/mL Dex plus LPS (1 μg/mL) for 24 h at 37 °C. Total protein was extracted from RAW264.7 cells with RIPA lysis buffer (CWBIO, Jiangsu, China) containing 1% protease inhibitor (CWBIO, Jiangsu, China) and 1% phosphatase inhibitor (CWBIO, Jiangsu, China), and the protein concentration was determined by a BCA protein assay kit (Elabscience, Wuhan, China). The lysate was boiled at 95 °C for 10 min, and the equal amount of protein (30 μg) was separated by 10% SDS–polyacrylamide gel electrophoresis and transferred to PVDF membranes (Millipore, Massachusetts, USA). To prevent non-specific binding, the membranes were blocked with 5% BSA solution (in Tris-buffered saline with 0.1% Tween 20) for 2 h at room temperature. The membranes were further incubated with primary antibodies against COX-2 (1:2000, Abcam), 5-LOX (1:1000, Abcam), Nrf2 (1:1000, Cell Signaling Technology), HO-1 (1:2000, Abcam), NQO1 (1:10,000, Abcam), p-NF-κB (1:1000, Cell Signaling Technology), p-IKKβ (1:1000, Affinity), p-IkBα (1:10,000, Abcam) and β-actin (1:3000, Proteintech) at 4 °C overnight. The membrane was washed with TBST (8 min × 3), followed by incubation with secondary antibodies (Rabbit IgG, 1:5000, Proteintech) for 1.5 h. After three washes with TBST, protein expression was detected using ECL reagent and the membrane was subsequently exposed to the imaging system (Amersham Imager 600, Massachusetts, USA). The intensity of protein bands was measured by optical density measurement and quantified by Image J software.

### Statistical analysis

Each experiment was repeated at least three times, and the data were expressed as mean ± standard deviation (SD). One-way ANOVA was conducted using GraphPad 8.0 software to determine the statistical significance between different groups, with *p* < 0.05 considered statistically significant.

## Supplementary Information

Supplementary Information.

## Data Availability

Data is provided within the manuscript or supplementary information files.
