# Supplementary material for: α-spinasterol isolated from Achyranthes aspera L. ameliorates inflammation via NF-κB and Nrf2/HO-1 pathways
Source: Sci Rep. 2025 Feb 17;15:5723. doi: 10.1038/s41598-025-90022-2 (PMC11833118; doi:10.1038/s41598-025-90022-2)
Supplement: Supplementary file 1 — Supplementary Information. [file 41598_2025_90022_MOESM1_ESM.pdf]

α-Spinasterol Isolated from *Achyranthes aspera* L. Ameliorates Inflammation via NF-κB and Nrf2/HO-1 Pathways

Qiongli Zeng[1, 2], Weiting Xiao[1, 2], Heng Zhang[1, 2], Wei Liu[1, 2], Xionglong Wang[4], Zheng Li[4], Yue Han[1, 2], Zhi Wang[1], Shunxiang Li[1], Jinwei Yang[3, 5*] & Wen Ouyang[1, 2, 5*]

1. Compounds Identification

1.1 Semi−Preparative High Performance Liquid Chromatography of Mixed Crystallization

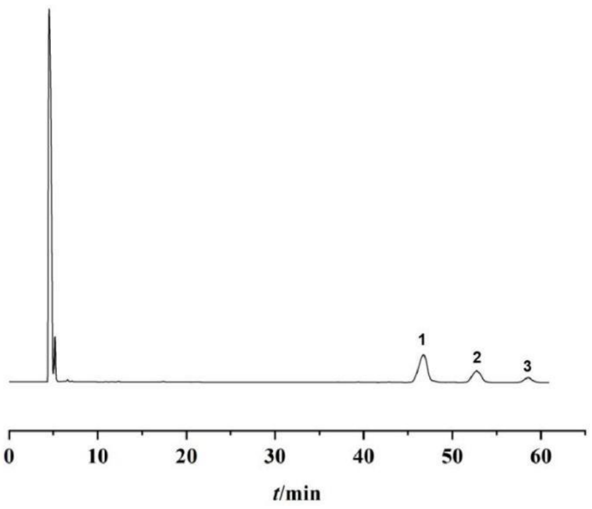

Fig. 1 The chromatogram of the three compounds. α-spinasterol (**1**), 22,23-dihydrospinasterol (**2**), 7,8-dihydrospinasterol (**3**)

1.2 Nuclear Magnetic Resonance Date

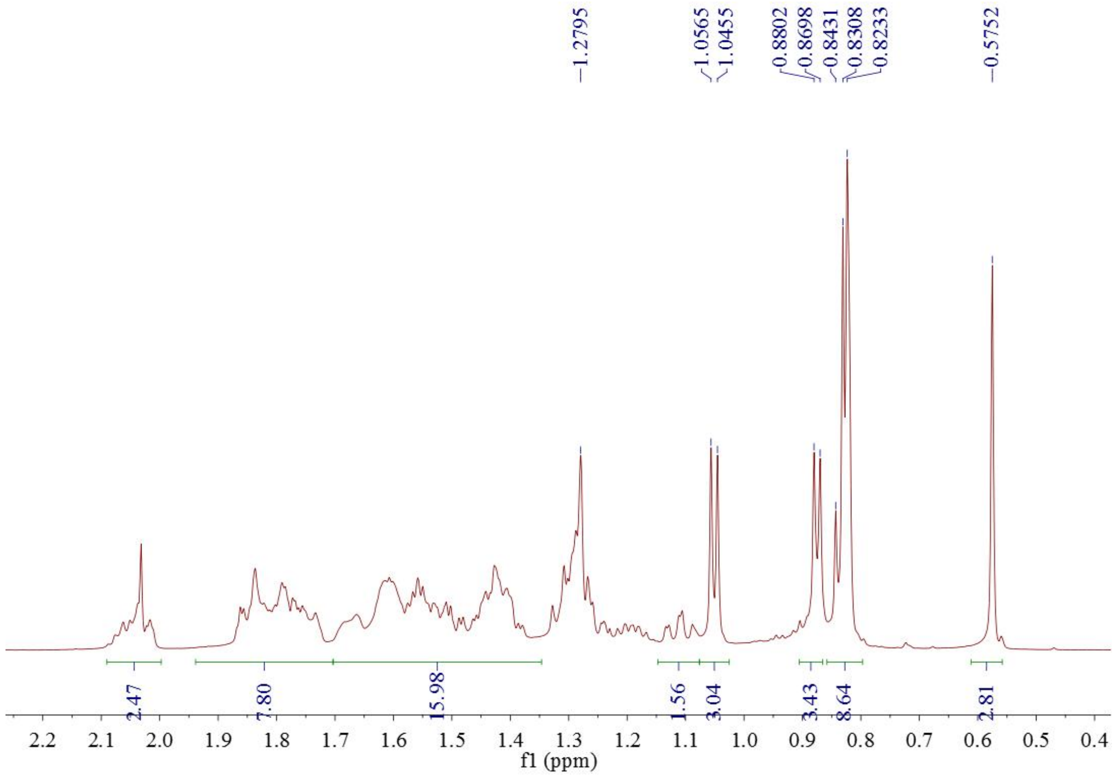

Fig. 2 ^{1}H-NMR of α-spinasterol (1)

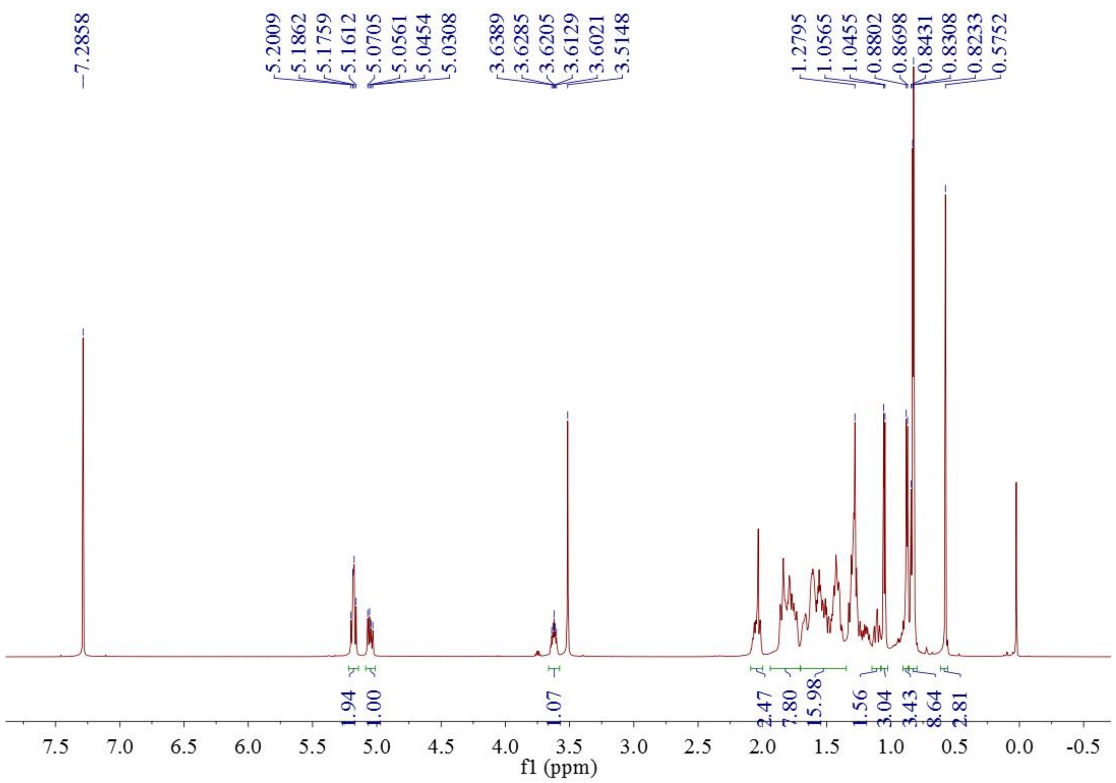

Fig. 3 ^{1}H-NMR of α-spinasterol (2)

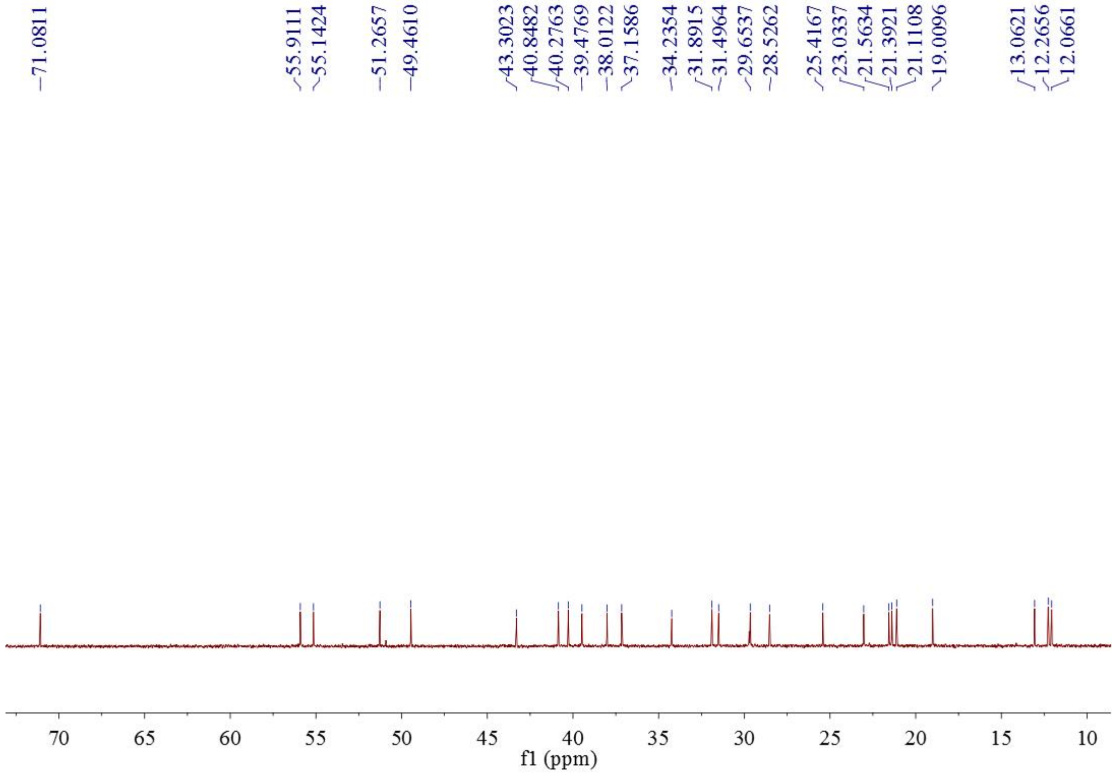

Fig. 4 ^{13}C-NMR of α-spinasterol (1)

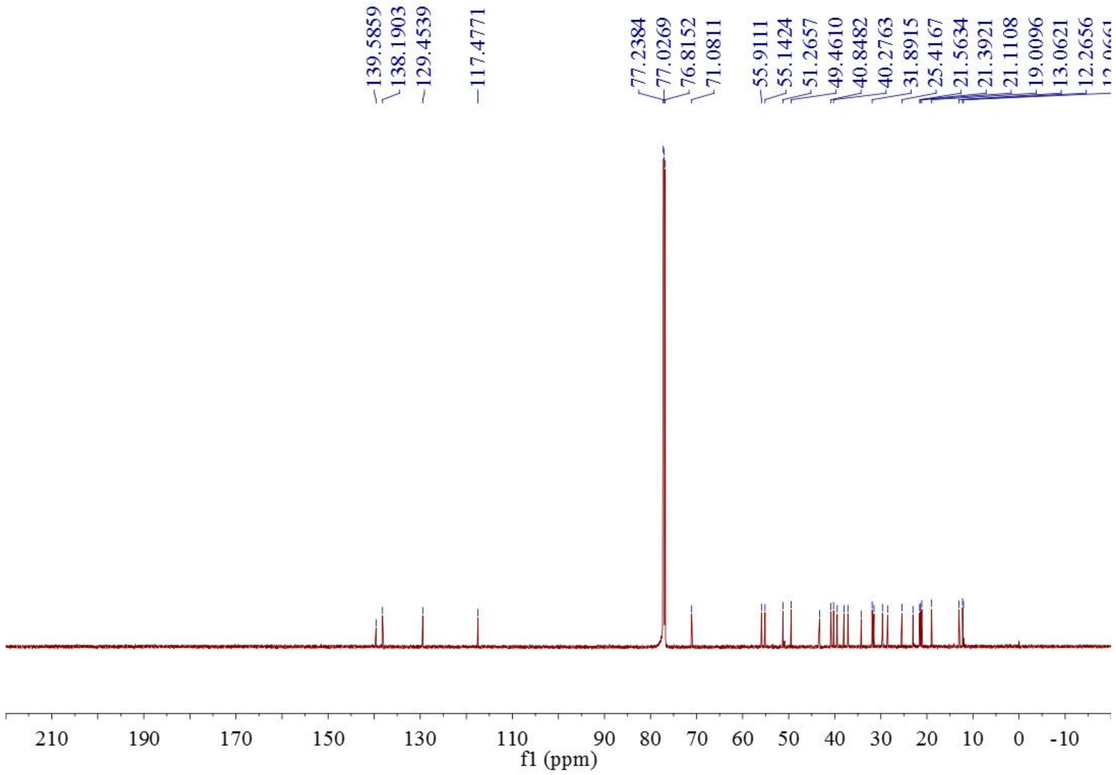

Fig. 5 ^{13}C-NMR of α-spinasterol (2)

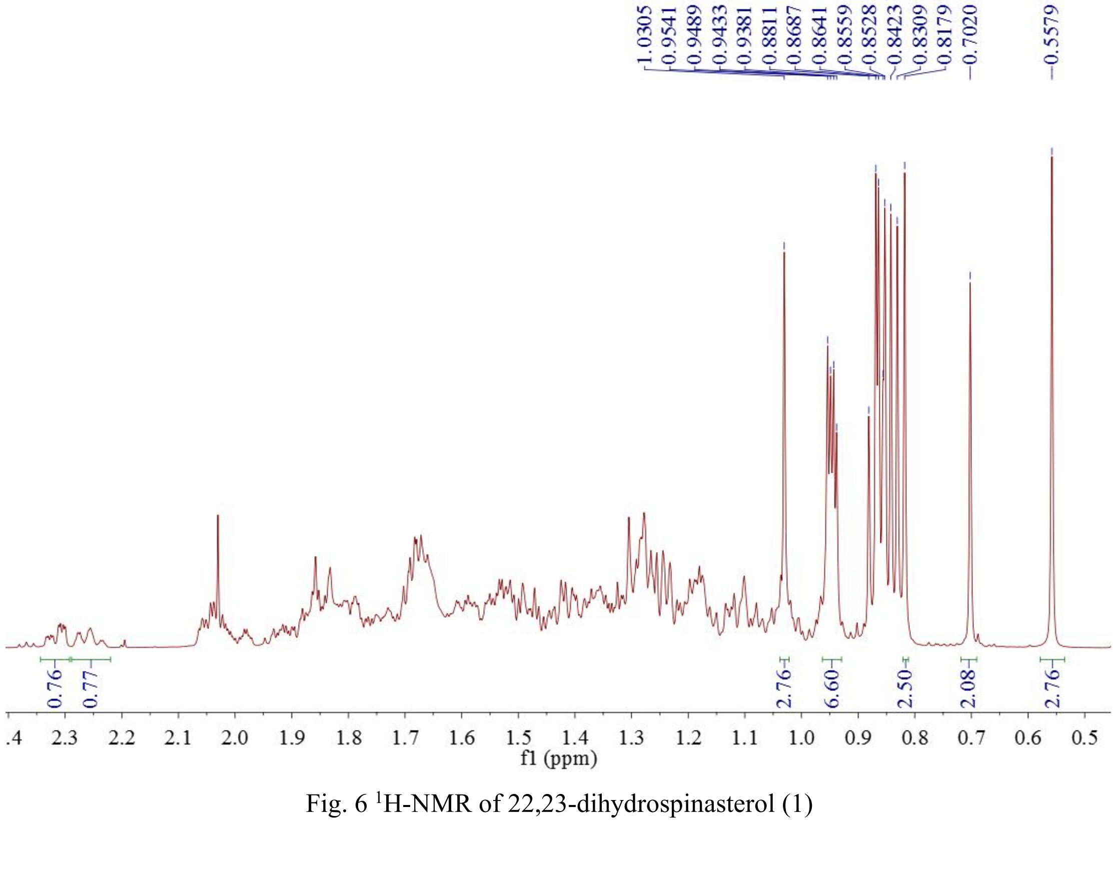

Fig. 6 ^{1}H-NMR of 22,23-dihydrospinasterol (1)

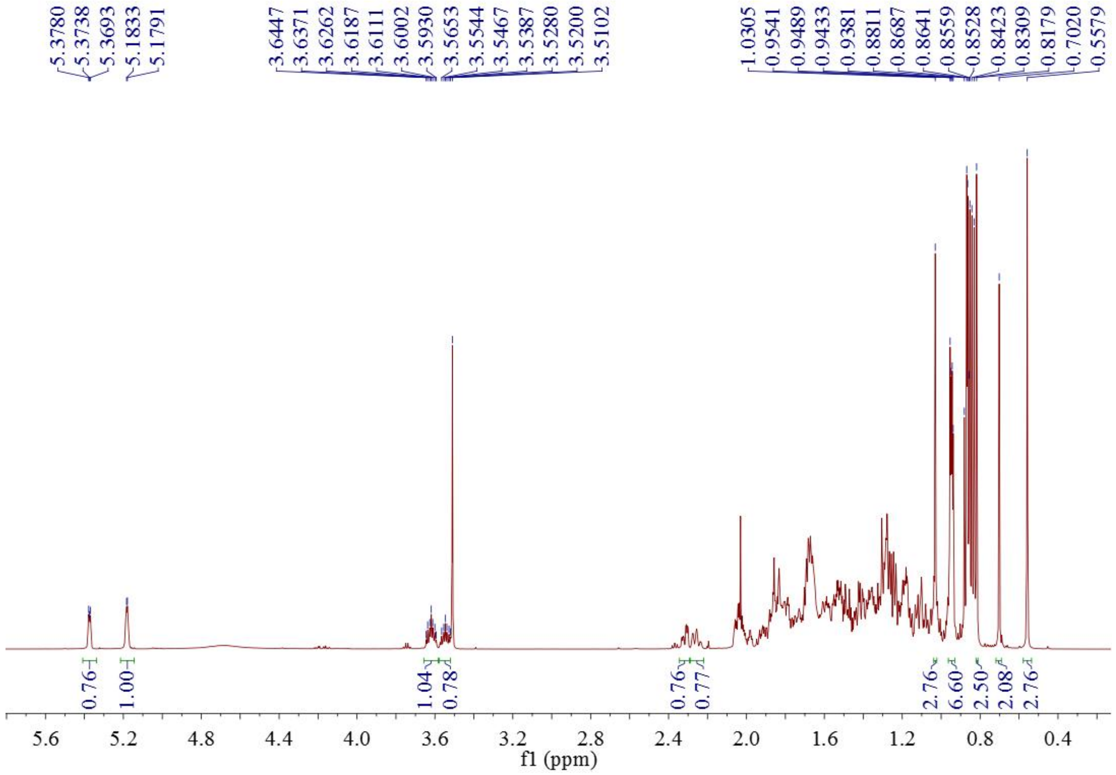

Fig. 7 ^{1}H-NMR of 22,23-dihydrospinasterol (2)

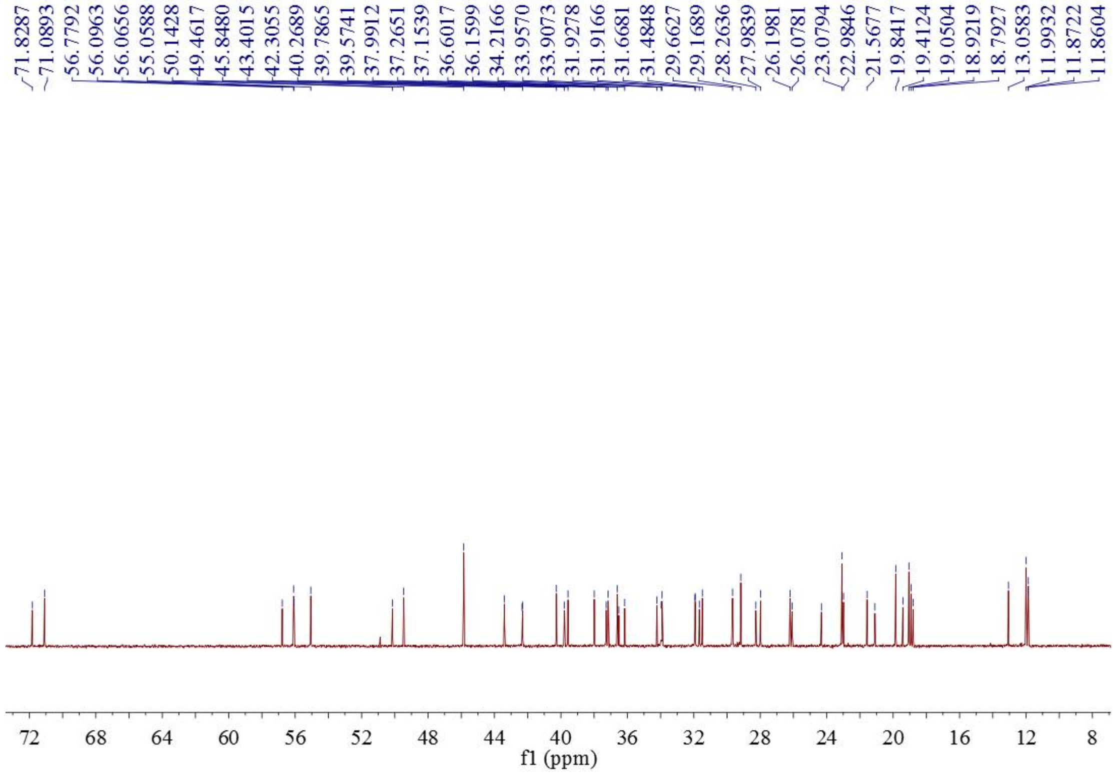

Fig. 8 ^{13}C-NMR of 22,23-dihydrospinasterol (1)

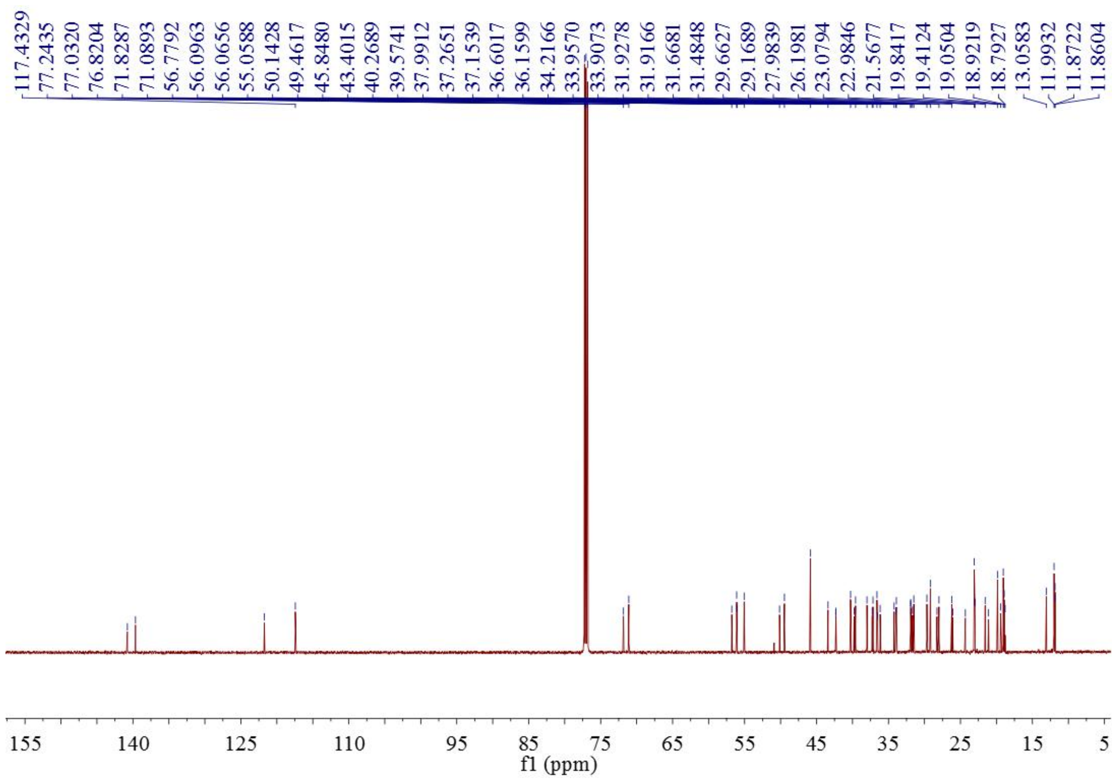

Fig. 9 ^{13}C-NMR of 22,23-dihydrospinasterol (2)

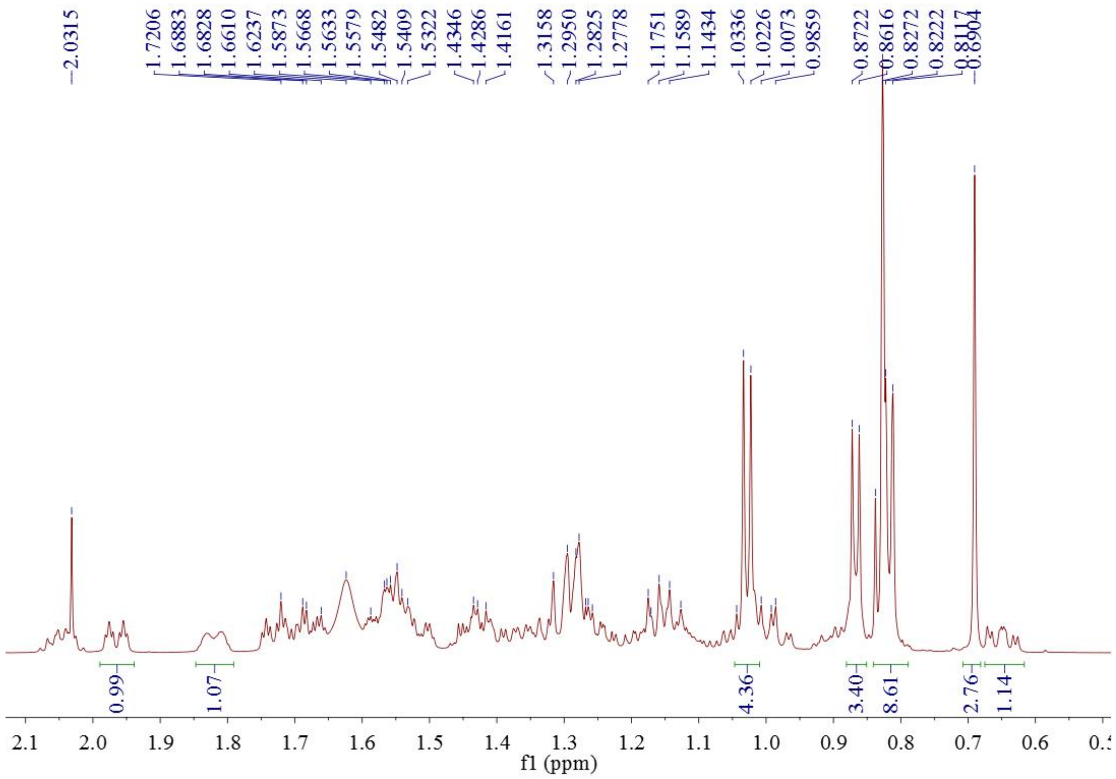

Fig. 10 ^{1}H-NMR of 7,8-dihydrospinasterol (1)

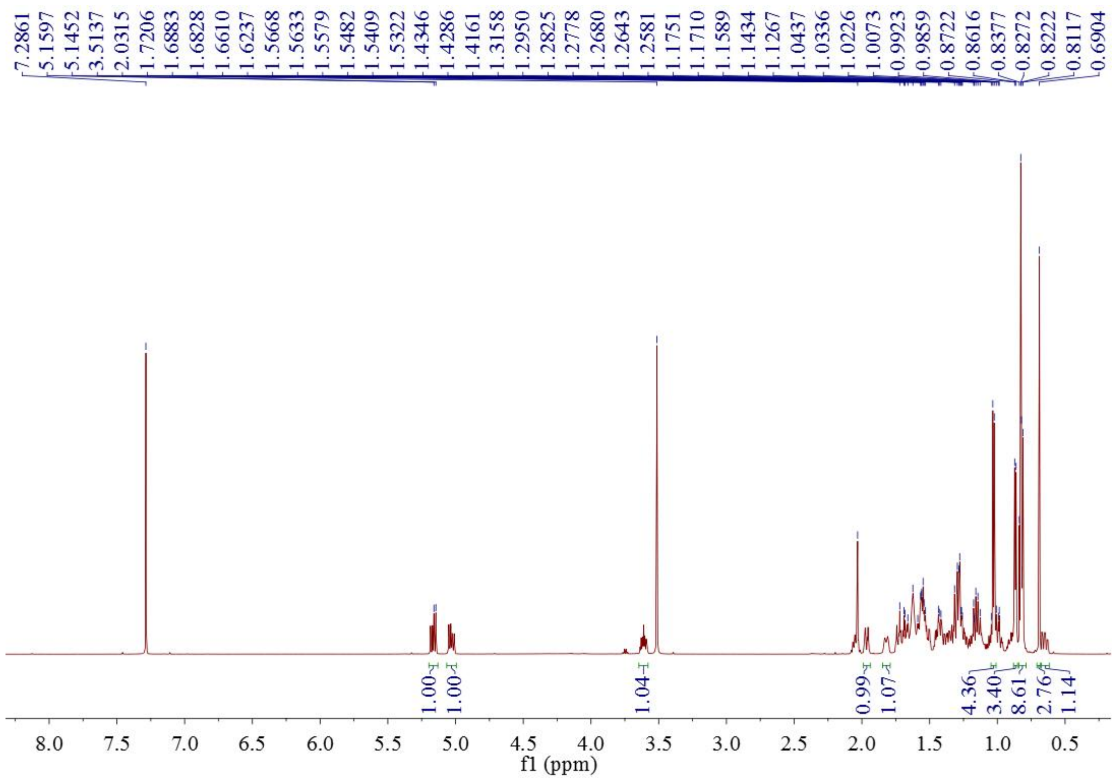

Fig. 11 ^{1}H-NMR of 7,8-dihydrospinasterol (2)

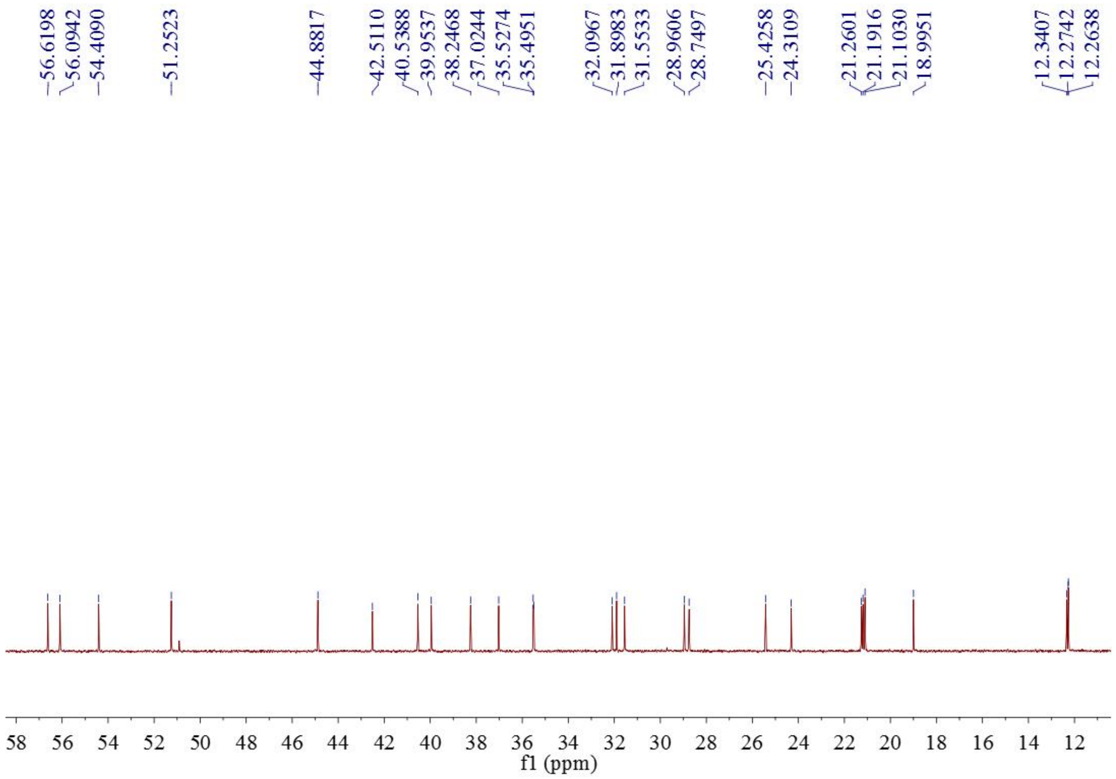

Fig. 12 ^{13}C-NMR of 7,8-dihydrospinasterol (1)

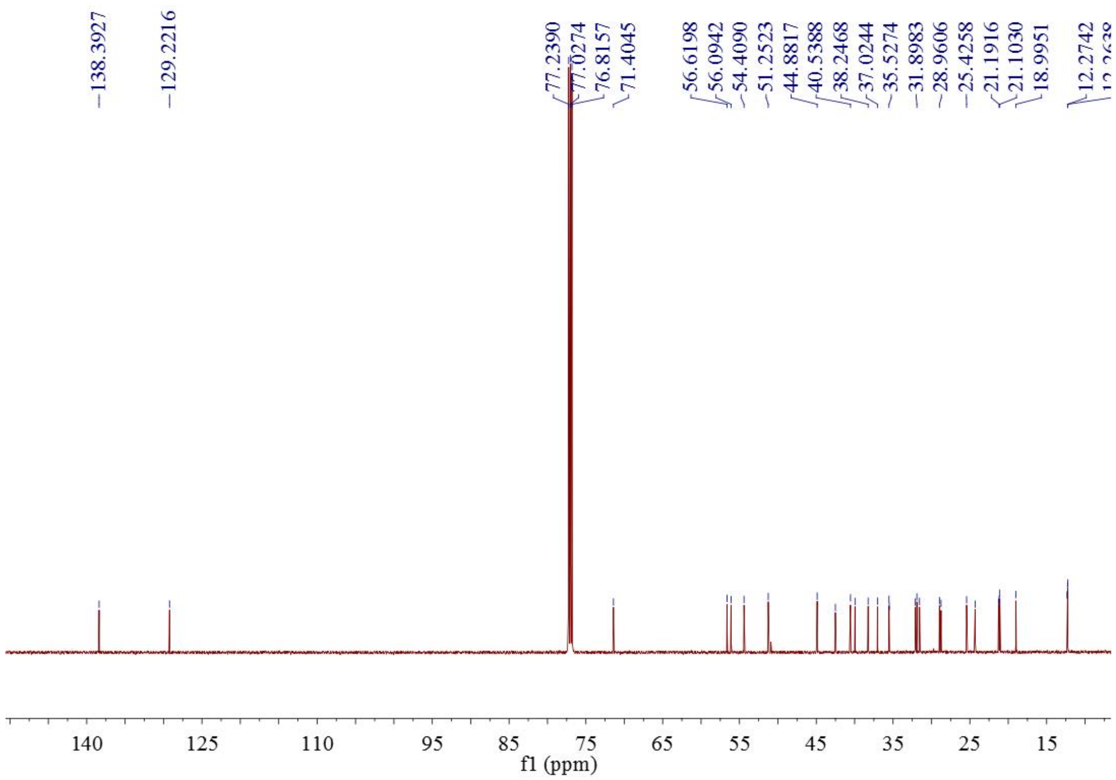

Fig. 13 ^{13}C-NMR of 7,8-dihydrospinasterol (2)

2. Determination of α-Spinasterol Content

2.1 HPLC Chromatograms of Standard and Sample

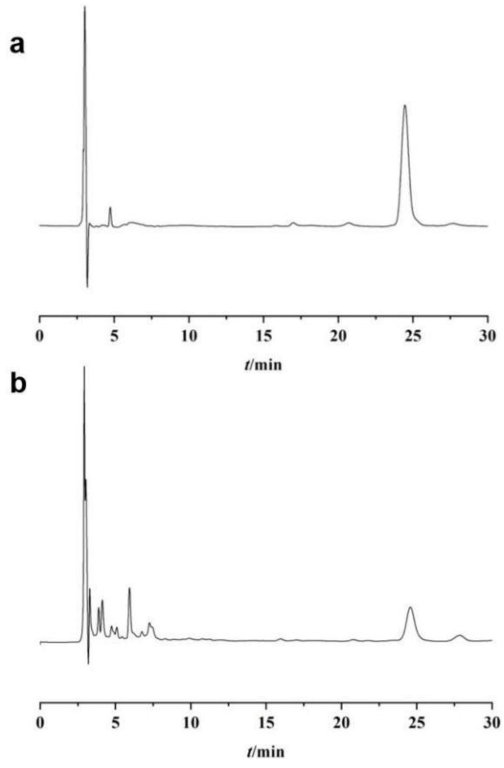

Fig. 14 (a) HPLC chromatograms of α-spinasterol standard. (b) HPLC chromatograms of *A. aspera*

2.2 Method validation of HPLC

Table 1 Regression equation, linear range and correlation coefficient of α-spinasterol

Regression equation	Range of linearity（mg/mL）	R^2
y = 348916x + 61193	0.025-0.5	0.9992

Table 2 Experimental results of recovery rate

Sample weight(mg)	Measured(mg)	Adding quantity (mg)	Recovery rate (%)	Average recoveries (%)	RSD(%)
4.0074	0.5931	0.4200	1.006	98.95	0.81
4.0078	0.5932	0.4200	1.010		
4.0108	0.5936	0.4200	1.007		
4.0052	0.5928	0.4200	1.013		
4.0179	0.5946	0.4200	1.013		
4.0007	0.5921	0.4200	1.004		

2.3 Content determination results of HPLC

Table 3 Changes of α-spinasterol content in *A. aspera* in different months

Months	Content (%)	standard deviation (%)	Average content (%)
4	0.0158	0.0005	0.0091±0.0003
5	0.0133	0.0004	
6	0.0064	0.0005	
7	0.0084	0.0005	
8	0.0090	0.0009	
9	0.0050	0.0002	
10	0.0050	0.0004	
11	0.0085	0.0004	
12	0.0110	0.0003	

3. Western Blot experimental results

3.1 α-Spinasterol's Effects on Proteins Expression of COX-2

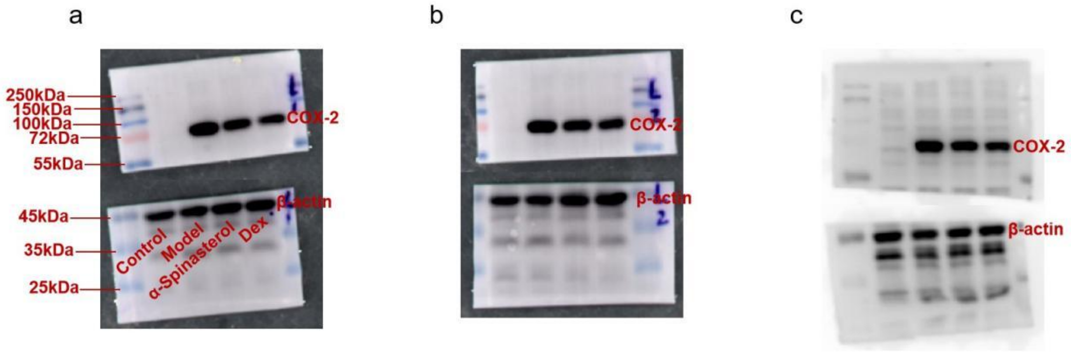

Fig. 15 α-Spinasterol's effects on proteins expression of COX-2 in induced RAW264.7 cells

3.2 α-Spinasterol's Effects on Proteins Expression of 5-LOX

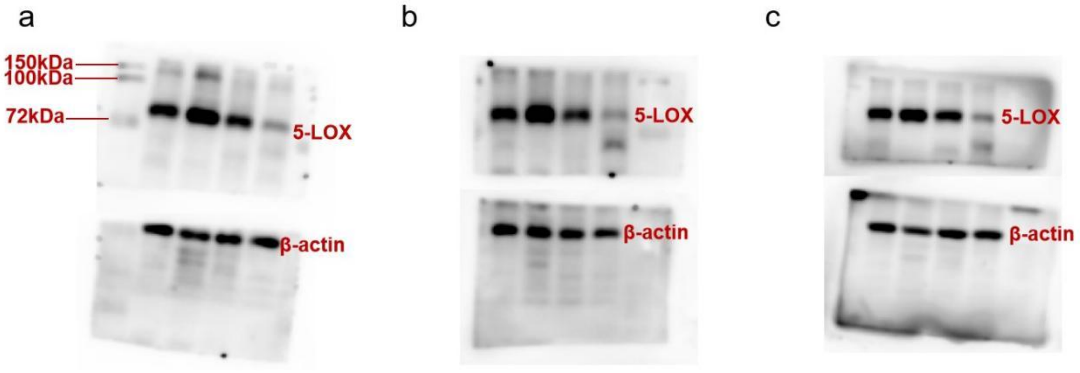

Fig. 16 α-Spinasterol's effects on proteins expression of 5-LOX in induced RAW264.7 cells

3.3 α-Spinasterol's Effects on Proteins Expression of p-IKKβ

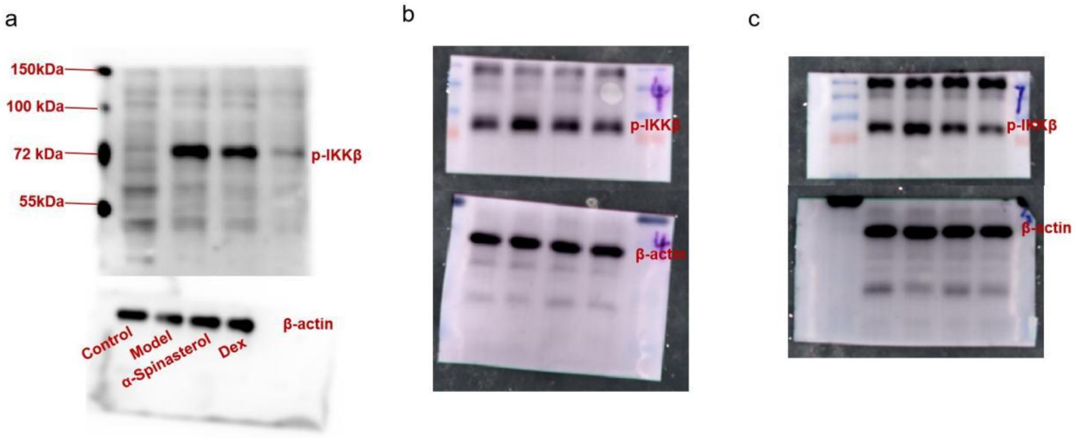

Fig. 17 α-Spinasterol's effects on proteins expression of p-IKKβ in induced RAW264.7 cells

3.4 α-Spinasterol's Effects on Proteins Expression of p-NFκB

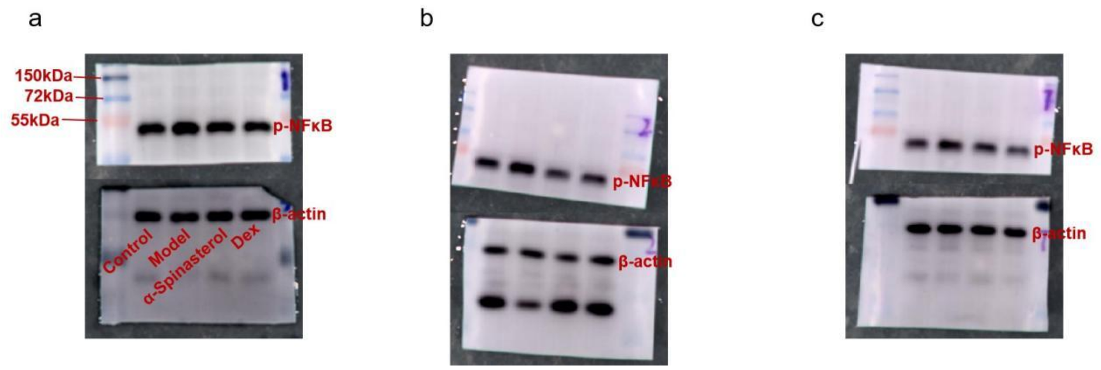

Fig. 18 α-Spinasterol's effects on proteins expression of p-NFκB in induced RAW264.7 cells

3.5 α-Spinasterol's Effects on Proteins Expression of p-IkBα

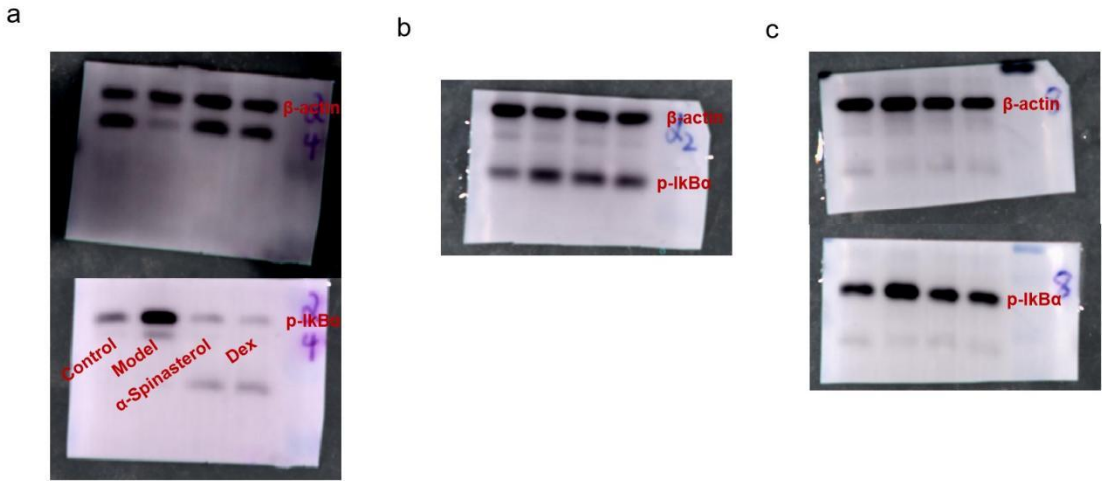

Fig. 19 α-Spinasterol's effects on proteins expression of p-IkBα in induced RAW264.7 cells

3.6 α-Spinasterol's Effects on Proteins Expression of Nrf2

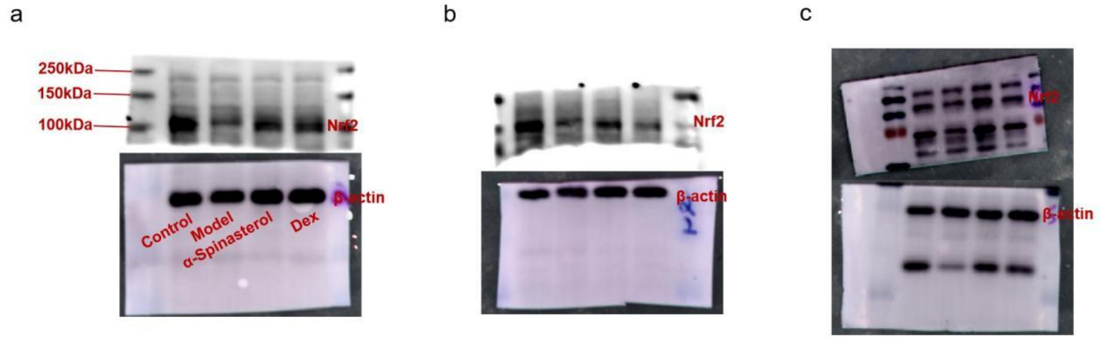

Fig. 20 α-Spinasterol's effects on proteins expression of Nrf2 in induced RAW264.7 cells

3.7 α-Spinasterol's Effects on Proteins Expression of HO-1

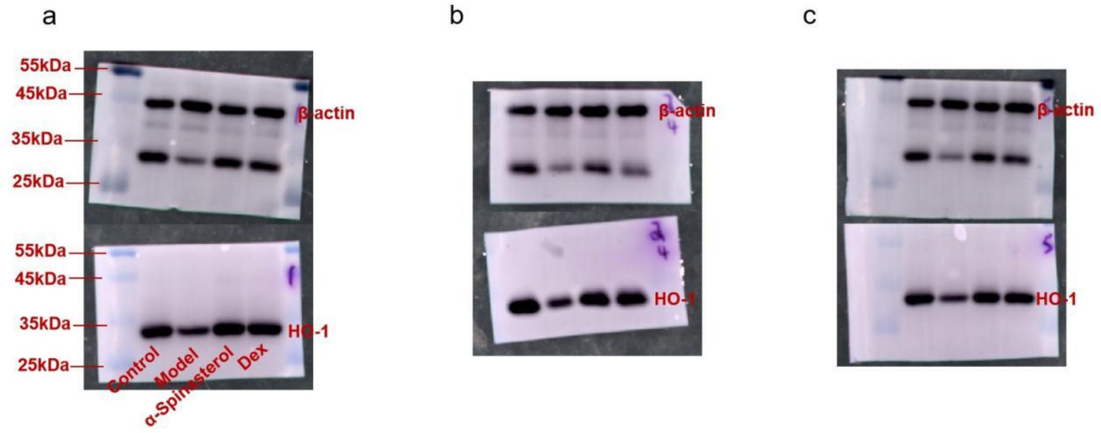

Fig. 21 α-Spinasterol's effects on proteins expression of HO-1 in induced RAW264.7 cells

3.8 α-Spinasterol's Effects on Proteins Expression of NQO1

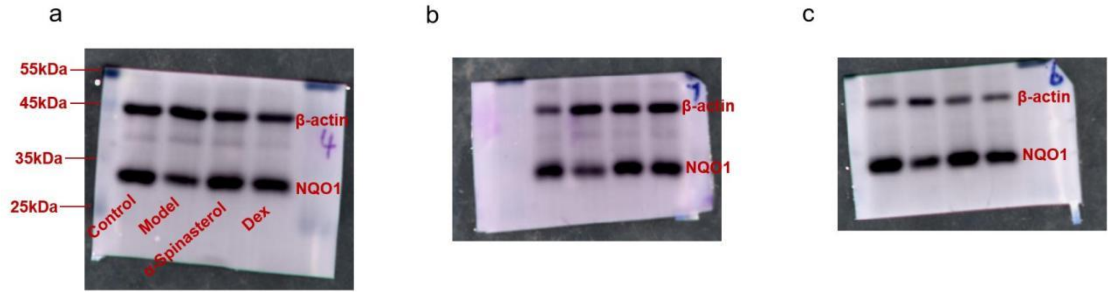

Fig. 22 α-Spinasterol's effects on proteins expression of NQO1 in induced RAW264.7 cells
